# Distinctive High Expression of Antiretroviral APOBEC3 Protein in Mouse Germinal Center B Cells

**DOI:** 10.3390/v14040832

**Published:** 2022-04-17

**Authors:** Shota Tsukimoto, Yoshiyuki Hakata, Sachiyo Tsuji-Kawahara, Takuji Enya, Tetsuo Tsukamoto, Seiya Mizuno, Satoru Takahashi, Shinichi Nakao, Masaaki Miyazawa

**Affiliations:** 1 Department of Immunology, Faculty of Medicine, Kindai University, 377-2 Ohno-Higashi, Osaka-Sayama 589-8511, Osaka, Japan; s-tsukimoto@med.kindai.ac.jp (S.T.); hakata@med.kindai.ac.jp (Y.H.); skawa@med.kindai.ac.jp (S.T.-K.); enyatakuji@yahoo.co.jp (T.E.); or tetsuo-tsukamoto@nuhw.ac.jp (T.T.); 2 Department of Anesthesiology, Faculty of Medicine, Kindai University, 377-2 Ohno-Higashi, Osaka-Sayama 589-8511, Osaka, Japan; nakaos@med.kindai.ac.jp; 3 Department of Pediatrics, Faculty of Medicine, Kindai University, 377-2 Ohno-Higashi, Osaka-Sayama 589-8511, Osaka, Japan; 4 Laboratory Animal Resource Center in Transborder Medical Research Center, Department of Laboratory Animal Science, Faculty of Medicine, University of Tsukuba, Tsukuba 305-8575, Ibaraki, Japan; konezumi@md.tsukuba.ac.jp; 5 Laboratory Animal Resource Center in Transborder Medical Research Center, Department of Anatomy and Embryology, Faculty of Medicine, University of Tsukuba, Tsukuba 305-8575, Ibaraki, Japan; satoruta@md.tsukuba.ac.jp; 6 Anti-Aging Center, Kindai University, 3-4-1 Kowakae, Higashiosaka 577-8502, Osaka, Japan

**Keywords:** cytidine deaminase, virus restriction, APOBEC3, protein expression, genome editing, tissue distribution, germinal center

## Abstract

Tissue and subcellular localization and its changes upon cell activation of virus-restricting APOBEC3 at protein levels are important to understanding physiological functions of this cytidine deaminase, but have not been thoroughly analyzed in vivo. To precisely follow the possible activation-induced changes in expression levels of APOBEC3 protein in different mouse tissues and cell populations, genome editing was utilized to establish knock-in mice that express APOBEC3 protein with an in-frame FLAG tag. Flow cytometry and immunohistochemical analyses were performed prior to and after an immunological stimulation. Cultured B cells expressed higher levels of APOBEC3 protein than T cells. All differentiation and activation stages of freshly prepared B cells expressed significant levels of APOBEC3 protein, but germinal center cells possessed the highest levels of APOBEC3 protein localized in their cytoplasm. Upon immunological stimulation with sheep red blood cells in vivo, germinal center cells with high levels of APOBEC3 protein expression increased in their number, but FLAG-specific fluorescence intensity in each cell did not change. T cells, even those in germinal centers, did not express significant levels of APOBEC3 protein. Thus, mouse APOBEC3 protein is expressed at distinctively high levels in germinal center B cells. Antigenic stimulation did not affect expression levels of cellular APOBEC3 protein despite increased numbers of germinal center cells.

## 1. Introduction

Apolipoprotein B mRNA editing enzyme, catalytic polypeptide-like 3 (APOBEC3) proteins are polynucleotide cytidine deaminases that belong to the activation-induced cytidine deaminase (AID)/APOBEC family proteins and have emerged in placental mammals through gene duplication and diversification which most likely occurred under a conflict with ancient retroviruses [1,2]. Humans possess seven APOBEC3 (A3) paralogues, A3A, A3B, A3C, A3D, A3F, A3G, and A3H, while mice have only a single A3-encoding locus in the haploid genome that shows genetic polymorphisms within this species [1,2,3]. A3 proteins function as restriction factors of diverse retroviruses and retrotransposable elements as well as of some other RNA and DNA viruses including enteroviruses, paramyxoviruses, coronaviruses, adenoviruses, human papillomavirus, hepatitis B virus, and Epstein-Barr virus (reviewed in [4,5]). A3 is known to restrict virus replication through its enzymatic activity in which the protein deaminates cytosine bases in single-stranded DNA and generates uracils, resulting in a high frequency of G-to-A substitutions in the complementary strand of the viral genome [3,4,5]. In the case of retroviruses, this deaminase-dependent restriction largely depends on incorporation of A3 proteins into newly budding virions and deamination of nascent minus-strand cDNA upon reverse transcription of viral genomic RNA in target cells of infection. A3 proteins can also exhibit antiviral functions through deaminase-independent mechanisms which include inhibition of reverse transcription, interference with proviral integration, and reduced processing and activities of viral protease (reviewed in [3,4]). However, to maintain efficient replication capacities to survive, retroviruses have acquired viral gene products that counteract host cell A3. Lentiviral Vif is an eminent example and has driven the evolution of primate A3 in the past millions of years (reviewed in [6]).

Despite possessing only a single A3 locus, mouse A3 (mA3) functions as a physiological restriction factor to mouse retroviruses including mouse mammary tumor virus (MMTV) [7], Friend murine leukemia virus complex (FV) [8,9], and AKR virus [10]. In fact, mA3 polymorphisms are associated with natural resistance or susceptibility to mouse retrovirus infection and disease development [8,9,11], and evidence indicates selective pressure for the evolution of mA3 polymorphisms in *Mus* species [12,13].

As A3 functions pleiotropic from the induction of tissue-specific gene expression and regulation of cell differentiation to retroelement restriction, its expression levels, and tissue and subcellular localization must be under strict physiological control, as dysregulated A3 expression can be associated with several diseases, such as tumorigenesis [14,15,16]. Tissue-specific expression and proper subcellular distribution of A3 are also important for effective protection of target cells from infection and prevention of virus persistence in potential reservoirs [15,17]. In this regard, protein level analyses of A3 expression and distribution, rather than mRNA expression analyses, are especially important as genetically determined resistance to mouse retrovirus infections is associated with high translation efficiency of the transcripts encoded by the highly restricting allele [13]. Further, human A3H haplotypes with differing protein stability [18] are associated with Vif adaptation in recently HIV-1-infected patients [19]. Only a limited number of studies have been performed to describe tissue and cellular localization of A3 proteins in humans and laboratory animals by utilizing polyclonal and some monoclonal antibodies (Ab). These indicate expressions of multiple A3 paralogue proteins in germinal centers of human adenoid and palatine tonsils at apparently lower levels than that of AID [20], constitutive expression of A3G, and induction of A3A protein upon IFN-α stimulation in human naive T cells [21], A3G expression in tumor-infiltrating human T cells [22] and epithelial cells of renal tubules in macaques [23]. Mouse A3 protein is expressed in spleen and mammary epithelial cells [24], primary keratinocytes [25] as well as in cultured embryonal fibroblasts [26]. However, most of these studies are descriptive, and detailed identities of cells expressing A3 proteins and the possible changes in expression levels and distribution of A3 proteins upon cell activation and/or virus infection are difficult to follow, especially because Ab generated to A3 proteins are often cross-reactive to other cellular proteins [13,26,27]. To overcome these limitations and to precisely follow the possible activation-induced changes in physiologically expressed levels of mA3 protein in different mouse tissues and specific cell populations, we utilized genome editing technology and generated knock-in (KI) strains of mice that express mA3 protein-tagged in-frame with FLAG peptide.

## 2. Materials and Methods

### 2.1. Mice

C57BL/6NCrSlc (B6) mice used at Kindai University were purchased from Japan SLC, Inc., Hamamatsu, Japan. All mice were housed and bred in the Experimental Animal Facilities at Kindai University Faculty of Medicine under specific pathogen-free conditions. Mice 7 to 20 weeks in age were used in this study. Mice used in Tsukuba University are described below.

### 2.2. Generation of Apobec3-FLAG KI Mice

**Figure 1:**
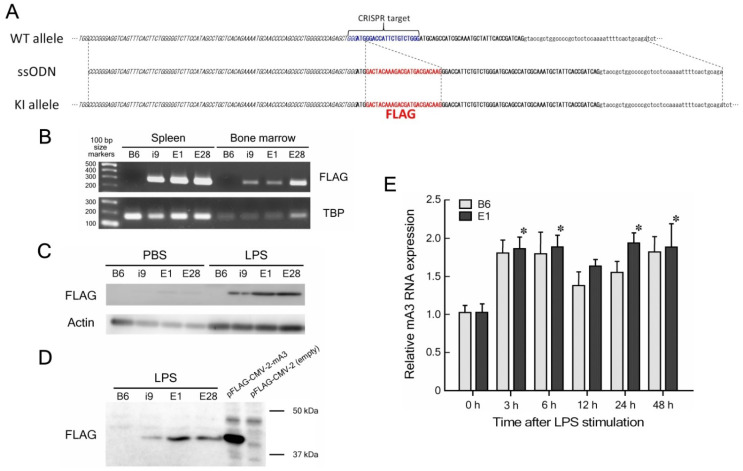
**Generation of KI animals that express FLAG-tagged mA3 protein.** (**A**) Nucleic acid sequences of the WT *Apobec3* allele in B6 mice, ssODN, and KI allele intended to be generated by CRISPR-Cas-9-mediated genome editing. Exon 1 untranslated region (UTR) is italicized and coding sequence (CDS) in bold face. Intron 1 is shown in lower case. CRISPR target sequence is indicated by blue and FLAG sequence by red. For more details, see Figure S1. (**B**) RT-PCR detection of FLAG-tagged mA3 mRNA from spleen cells. TATA-binding protein (TBP) mRNA was detected as positive control. Pictures of the entire agarose gel are shown in Figure S2. Experiments were performed twice with essentially identical results. (**C**) Western blot assays detecting FLAG-tagged mA3 protein from cultured spleen cells. Spleen cells were cultured for 2 days either in the absence (PBS) or presence of LPS, and whole cell proteins were extracted with the lysis buffer and dissolved in SDS-PAGE sample buffer. Actin was used as loading control. Pictures of the entire blotted membrane are shown in Figure S3. Experiments were performed twice with essentially identical results. (**D**) Western blot comparison of apparent molecular mass of the FLAG-tagged protein expressed in LPS-stimulated KI spleen cells with that expressed in 293T cells transfected with the pFLAG-CMV-2 plasmid harboring the mA3 cDNA [26]. A photograph of the entire blotted membrane is shown in Figure S4. Experiments were performed twice with essentially identical results. (**E**) Real-time RT-PCR for quantification of mA3 mRNA in cultured spleen cells at different time-points after LPS stimulation. Spleen cells were prepared from three mice for each genetic group, and cells from each individual mouse were cultured in a separate set of wells to prepare mRNA at the indicated time-points. Amounts of mA3 mRNA relative to the average of three samples cultured without LPS for each genetic group are shown. Bars indicate mean + SEM (*n* = 3). Two-way repeated measure ANOVA indicated no significant differences between the two genetic groups (column factor *p* = 0.0967), while there was a significant time-wise difference (*p* = 0.0025). Separate one-way ANOVA comparisons of all time-points in each genetic group revealed significant increase of mA3 mRNA expression from that before stimulation at 3, 6, 24 and 48 h after LPS stimulation in E1 spleen cells (* *p* < 0.05). Similar increase in mA3 mRNA expression upon LPS stimulation in both WT B6 and E1 mice were confirmed in a separate experiment. Cloning of the mA3 cDNA from E1 spleen cells and its sequencing confirmed complete march with the consensus isoform 2 sequence (Figure S5).

The *Apobec3*-FLAG KI mice were generated by CRISPR-Cas9-based genome editing as described previously [28,29]. A sequence (5′-GGG ATG GGA CCA TTC TGT CT-3′) was selected as the guide RNA (gRNA) target. We designed a 200-nt single-stranded oligodeoxynucleotide (ssODN) donor in which the FLAG tag sequence was placed between 88-nt 5′ and 88-nt 3′ homology arms (Figure 1A). Only a single FLAG tag sequence was inserted in-frame in the N-terminus of mA3 polypeptide as secondary structure prediction with JPred4 [30] indicated formation of an additional α-helix in the N-terminus of mA3 protein if three tandem repeats of FLAG peptide were inserted. This ssODN was ordered from Integrated DNA Technologies (Coralville, IA, USA) as Ultramer DNA oligonucleotide. 

We attempted to generate KI mice by microinjection and electroporation. For microinjection, the gRNA target sequence described above was inserted into the entry site of the pX330 plasmid (Addgene plasmid #42230), which has both gRNA and Cas9 expression units, to construct the pX330-*Apobec3* vector. This plasmid was isolated from transformed *E. coli* cells with FastGene Plasmid Mini Kit (Nippon Genetics, Tokyo, Japan) and filtrated through 0.22 μm MILLEX-GV filter (Merk Millipore, Darmstadt, Germany). The pregnant mare serum gonadotropin (5 units) (ASKA Animal Health, Tokyo, Japan) and the human chorionic gonadotropin (5 units) (ASKA Animal Health) were intraperitoneally injected into female C57BL/6J mice (Charles River Laboratories, Kanagawa, Japan) with a 48-h interval, and mated with male C57BL/6J mice. We collected zygotes from oviducts in mated female and a mixture of pX330-*Apobec3* (circular, 5 ng/μL) and ssODN (10 ng/μL) was microinjected into male pronucleus of the zygotes. Subsequently, surviving microinjected zygotes were transferred into oviducts in pseudopregnant female ICR mice (Charles River Laboratories). For electroporation, the gRNA was synthesized and purified by the GeneArt Precision gRNA Synthesis Kit (Thermo Fisher Scientific, Waltham, MA, USA) and dissolved in Opti-MEM medium (Thermo Fisher Scientific). Female C57BL/6J mice were superovulated in the same manner as described above, and unfertilized oocytes were collected from their oviducts. We then performed in vitro fertilization with these oocytes using sperms obtained from male C57BL/6J mice according to standard protocols. Five hours later, the mixture of gRNA (25 ng/μL), ssODN (100 ng/μL), and GeneArt Platinum Cas9 Nuclease (Thermo Fisher Scientific, 100 ng/μL) was electroporated to the zygotes by using the NEPA 21 electroporator (NEPAGENE, Tokyo, Japan) as described [29]. One day later, the electroporated embryos that had developed to the 2-cell stage were transferred into the oviducts of pseudopregnant female ICR mice and newborns were obtained. Genomic DNA sequences around Exon 1 of the *Apobec3* gene were determined as described [31] using the following primer: 5′-TAGGCAGGTTGGATGGAGAC-3′.

### 2.3. DNA Extraction and Genotyping

Tail and splenocyte DNA were extracted and purified essentially as described previously [9,32] using KAPA Mouse Genotyping Kit (KAPA Biosystems, Inc., Wilmington, MA, USA) according to the manufacturer’s protocol. Detection of the FLAG-tagged *Apobec3* allele was performed by polymerase chain reaction (PCR) using the following primer set: forward, 5′-TAGGCAGGTTGGATGGAGAC-3′, reverse, 5′-GGTGGCCAGTTTAGAAGCAG-3′, and internal, 5′-CTTGTCGTCATCGTCTTTGTAGT-3′. To distinguish homozygous from heterozygous animals the following primer set was used: forward, 5′-CAGAAAATGCAACCCCAGCGC-3′; reverse, 5′-TGAATAGCATTTGCGATGGCTGC-3′. Agarose gel electrophoresis and DNA band detection were done as described [13].

### 2.4. RNA Extraction, Reverse Transcription (RT)-PCR Assays and cDNA Sequencing

RNA was extracted from mouse spleen and bone marrow cells with RNeasy Plus Mini Kit (QIAGEN N.V., Venlo, The Netherlands), and reverse transcribed with SuperScript III First-Strand Synthesis System for RT-PCR (Invitrogen, Thermo Fisher Scientific). The RT products were amplified by using Taq DNA polymerase (Takara Bio, Inc., Kusatsu, Shiga, Japan) with the following primer set: forward (FLAG-specific), 5′-GACTACAAAGACGATGACGACAAG-3′; reverse, 5′-GCTAATACGACTCACTATAGGGAACAGCCAGTATAAAAAGCAGATTTCAGCGTGG-3′ to evaluate the expression of FLAG-tagged mA3 mRNA. For the amplification of TATA-binding protein (TBP) cDNA, primers designed for quantitative real-time RT-PCR as described below were utilized.

For cDNA sequencing, spleen cell RNA was prepared and reverse transcribed as described above, and the *Apobec3* cDNA was amplified by using primers specific for 5′ and 3′ UTR of the FLAG-tagged mA3 allele (5′-CAGAAAATGCAACCCCAGCGC-3′ and 5′-CATGCACAACTTAATCTTGTCTTTC-3′, respectively) and Pfu Turbo DNA Polymerase (Agilent Technologies, Santa Clara, CA, USA). The amplified fragment was cloned into pCR-Blunt II-TOPO plasmid using the Zero Blunt TOPO PCR Cloning Kit (Invitrogen) and sequenced as described [9].

### 2.5. Quantitative Real-Time RT-PCR

The above purified RNA was reverse transcribed using PrimeScript RT reagent Kit (Perfect Real Time, Takara Bio, Inc.). The resultant cDNA was amplified by using TB Green Premix Ex Taq II (Tli RNaseH Plus, Takara Bio, Inc.) with primers specific for mA3 and TBP (Takara Perfect Real Time Support System, primer set ID MA099660 for mA3 and MA050367 for TBP) using Step One Plus System (Applied Biosystems, Thermo Fisher Scientific). The results were analyzed by the 2^−∆∆Ct^ method.

### 2.6. Cell Culture

Spleen cell suspension was prepared as described [33], red cells lysed by incubating with Tris-buffered ammonium sulfate solution [34], washed with phosphate-buffered saline (PBS), and resuspended in RPMI 1640 medium supplemented with 10% fetal bovine serum (Sigma-Aldrich Co., LLC, St. Louis, MO, USA). These spleen cells were seeded at 3.0 × 10^6^ cells per well in 24-well plates and cultured either in the presence or absence of 1 μg/mL lipopolysaccharide (LPS 026:B6, Sigma-Aldrich Co., LLC).

### 2.7. Western Blotting

Western blotting analyses were conducted as described previously [13,26] with some modifications. Briefly, proteins were extracted from 1.0 × 10^6^ cells with a lysis buffer (50 mM Tris-HCl pH 8.0, 150 mM NaCl, 0.5% Triton X-100, and 2 mM EDTA) containing complete ULTRA protease inhibitor cocktail (Roche Applied Science, Mannheim, Germany). The extracts were mixed with sodium dodecyl sulfate (SDS)-polyacrylamide gel electrophoresis (PAGE) sample buffer and boiled for 5 min. The proteins were separated by SDS-PAGE, transferred to Immobilon-P membrane (Merck Millipore), and blotted membranes were blocked with Tris-buffered saline containing 0.05% Tween 20 (Takara Bio, Inc.) (TBS-T). The blocked membranes were incubated at room temperature either with the following anti-FLAG Ab for 2 h or with the anti-actin Ab for 1 h. Membranes were then washed with TBS-T and incubated with horse radish peroxidase (HRP)-conjugated secondary Ab of the appropriate specificity for 1 h at room temperature, washed again with TBS-T, and the bound Ab were detected using Luminata Forte Western HRP substrate (Merck Millipore). The images were captured with the ImageQuant LAS 4000 system (GE Healthcare, Chicago, IL, USA). Anti-FLAG rabbit monoclonal Ab (mAb) (D6W5B; Cell Signaling Technology, Inc., Danvers, MA, USA) and anti-actin mouse mAb (C4; Merck Millipore) were diluted at 1:2000 and 1:1000, respectively, with TBS-T and used as primary Ab. HRP-conjugated goat anti-rabbit and anti-mouse IgG Ab (Invitrogen) were diluted at 1:3000 and used as secondary Ab.

### 2.8. Immunohistochemistry

Immunohistochemical staining of fresh frozen sections were performed essentially as described previously [35] with some modifications. Briefly, freshly collected organs were embedded in OCT compound (Sakura Finetek Japan Co., Ltd., Tokyo, Japan) and frozen in liquid nitrogen. The frozen blocks were cut into sections of 4 μm in thickness using a CM3050S cryostat (Leica Biosystems Nussloch GmbH, Wetzlar, Germany), and placed on CREST COAT glass slides (Matsunami Glass Ind., Ltd., Kishiwada, Japan). Fixation was performed with a mixture of ethanol and acetone. To prevent non-specific binding of Ab, died tissue sections were incubated at room temperature for 1 h in a solution containing anti-Fc receptor (CD16/CD32) rat mAb (clone 2.4G2, IF Formulated; Cell Signaling Technology, Inc.) mixed with 10% unimmunized goat serum in PBS. The rabbit anti-FLAG mAb (D6W5B) was diluted 1:400 with PBS containing 10% unimmunized goat serum and sections were incubated with this Ab solution at 4 °C overnight. Goat anti-rabbit IgG (#40925, Invitrogen) was used as the secondary Ab for the detection of bound anti-FLAG mAb along with Alexa Fluor 594 Tyramide SuperBoost Kit (Invitrogen). Anti-mouse CD3 (17A2; eBioscience, Thermo Fisher Scientific) and anti-mouse B220 (CD45R) (RA3-6B2; eBioscience) mAb labeled with fluorescein isothiocyanate (FITC) or Alexa Fluor 488 were used to detect lymphocyte subsets. These Ab were also diluted with PBS containing 10% unimmunized goat serum and reacted with tissue sections at 4 °C overnight. PBS containing 0.05% Tween 20 (Takara Bio, Inc.) was used for washing in all steps. After staining, the sections were covered by using Fluoro-KEEPER Antifade Reagent, Non-Hardening Type with DAPI (Nacalai tesque, INC., Kyoto, Japan) and observed with an all-in-one fluorescence microscope BXZ-X800 (KEYENCE, Osaka, Japan).

### 2.9. Flow Cytometry

Flow cytometry was performed as described previously [32] with some modifications. The following two panels of mAb were used. Panel 1: anti-FLAG (D6W5B), anti-mouse CD3, and anti-mouse CD19 mAb conjugated, respectively, with phycoerythrin (PE), PerCP-Cy5.5, and BUV395; panel 2: anti-mouse Gr-1/anti-mouse CD11b conjugated with BV510, anti-mouse CD3 conjugated with BV711, anti-mouse CD93 conjugated with PE-Cy7, anti-mouse B220 conjugated with BB700, anti-mouse CD23 conjugated with BV421, anti-mouse IgM conjugated with BV650, anti-mouse CD19 conjugated with BUV395, anti-mouse CD21 conjugated with allophycocyanin (APC), anti-mouse IgD conjugated with BV785, anti-mouse GL7 conjugated with FITC, anti-mouse CD95 conjugated with BUV737, and the anti-FLAG mAb conjugated with PE (all purchased from Cell Signaling Technology, Inc.). For the detection of intracellular mA3 protein, cells were first incubated with the above mAb reactive with cell-surface molecules, washed, and then fixed and permeabilized by using Foxp3/Transcription Factor Staining Buffer Kit (Tonbo Biosciences, San Diego, CA, USA) according to the manufacturer’s instructions before incubating with the anti-FLAG mAb. Forward and side scatter and fluorescence signals were collected with BD LSRFortessa (BD Biosciences, Franklin Lakes, NJ, USA), and obtained data were analyzed with Flowjo (version10.6.2, BD Biosciences).

### 2.10. Immunization with Sheep Red Blood Cells (SRBC)

One mL of citrated sheep blood (Nippon Bio-Supp. Center, Tokyo, Japan) was washed twice with 50 mL PBS and resuspended at 1:10 in PBS (0.4 mL packed SRBC and 3.6 mL PBS). 100 µL of the above SRBC suspension was injected intravenously into wild-type (WT) B6 and mA3 FLAG-KI mice. Mice were killed by cervical dislocation under deep anesthesia 10 days after the above immunization and spleens were collected for flow cytometric analyses as described above.

### 2.11. Statistical Analyses

Data analyses were performed by using GraphPad Prism 5 (GraphPad Software, Inc., San Diego, CA, USA) with an appropriate method and post hoc tests for multiple comparisons where required. The method of analysis used and obtained *p* values are shown in each figure legend.

## 3. Results

### 3.1. Establishment of mA3 FLAG-KI Lines of Mice

FLAG KI at the mouse *Apobec3* gene locus was attempted either by microinjecting a plasmid expressing the gRNA and Cas9 along with the ssODN into the pronucleus of a fertilized egg or by electroporation of fertilized eggs to introduce the gRNA, Cas9 enzyme, and ssODN (Figure 1A). Sixty-three and 47 pups were obtained from microinjected and electroporated eggs, respectively, and tail DNA sequencing revealed that one male obtained through microinjection and one each male and female obtained through electroporation possessed the intended KI allele (Figure S1). These KI animals were mated with WT B6 mice and heterozygotes possessing the KI and WT alleles were used for further breeding and the following phenotype assessment. The KI line derived from the microinjected egg was designated i9 and those derived from electroporated eggs E1 and E28.

We first examined the expression of FLAG-tagged mA3 mRNA in the spleen and bone marrow (BM) by RT-PCR with the FLAG-specific primer set (Figure 1B). As expected, all three KI lines expressed FLAG-tagged mA3 mRNA both in the spleen and BM, and higher levels of mA3 mRNA expression in the spleen than in BM were consistent with our previous data [32]. We next attempted to detect the expression of FLAG-tagged mA3 protein by Western blot assays using spleen cells. As the base-line expression level of mA3 protein in mouse tissues is not high, we stimulated cultured spleen cells with LPS which has been shown to induce higher levels of mA3 expression [36,37]. As a result, the expression of FLAG-tagged mA3 protein in spleen cells cultured without LPS was barely detectable, while LPS stimulation vastly increased the levels of FLAG-tagged mA3 protein expression in all three KI lines (Figure 1C). To confirm that the FLAG-tagged protein detected from KI mouse spleen cells shows the molecular mass consistent with its being mA3, lysates of cultured 293T cells that are transfected either with the pFLAG-CMV-2 plasmid expressing mA3 or the empty vector were included in a separate experiment (Figure 1D). The FLAG-tagged protein detected from all three KI lines showed the apparent molecular mass identical to that of the positive control FLAG-tagged mA3, which was consistent with the molecular mass of the exon 5-lacking isoform reported previously [9].

The level of mA3 protein expression in line i9 was lower than those in the other two lines in repeated experiments, and while line E28 showed mA3 mRNA expression levels as high as those in line E1, this line bred poorly. Thus, line E1 was selected for further breeding, generation of homozygous KI individuals, and the following experiments. Analyses using quantitative real-time PCR showed similar kinetics in the increase in mA3 mRNA expression at 3–6 h and 1–2 days after LPS stimulation in both WT B6 and line E1 spleen cells (Figure 1E) with no significant group-wise difference, indicating that the mechanisms regulating mA3 gene expression were not apparently affected by the KI modification. We further cloned and sequenced the *Apobec3* cDNA from E1 spleen cells and confirmed that the coding sequence downstream of the FLAG insertion completely matched with the consensus sequence of mouse *Apobec3* isoform 2 that lacks exon 5 (Figure S5). Thus, these results have collectively indicated that the KI line of mice expresses FLAG-tagged mA3 protein of the expected amino acid sequence, and its expression is not affected by the insertion of the FLAG sequence.

### 3.2. Expression of mA3 Protein in LPS-Stimulated Spleen Cells

**Figure 2:**
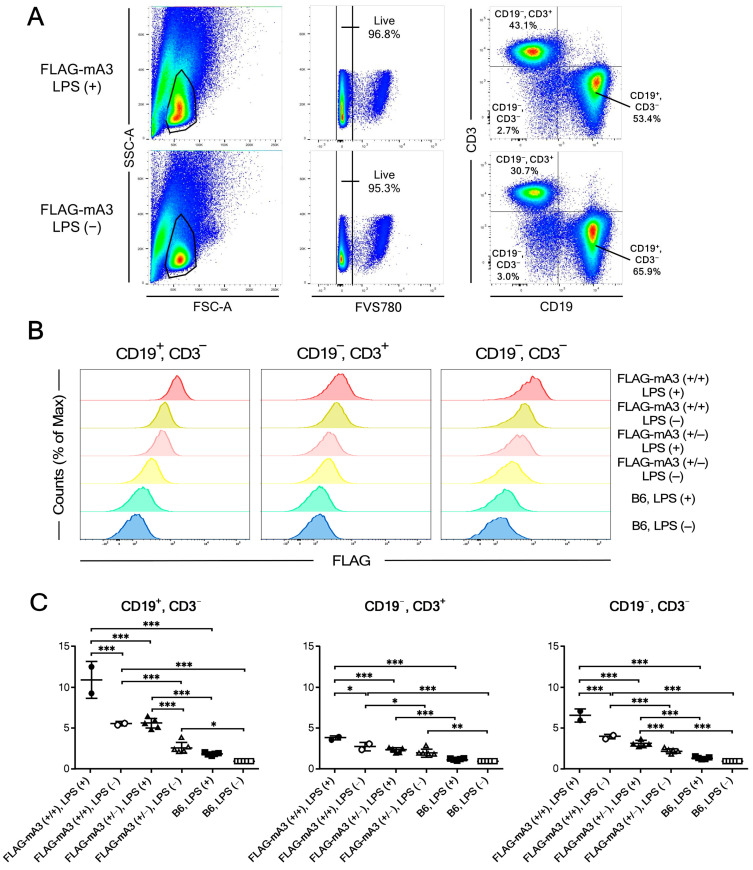
**Flow cytometric analyses of intracytoplasmic mA3 expression in different subpopulations of spleen cells after culturing with or without LPS.** (**A**) Representative gating strategies. Lymphoid cells were gated by using dot plots showing forward and side scatter areas (FSC-A and SSC-A, respectively), and dead cells were eliminated by staining with Fixable Viability Stain (FVS) 780. CD19^+^ B, CD3^+^ T, and non-B, non-T cells were gated by using dot plots showing fluorescence intensities of CD3 and CD19. One million events were used to generate shown dot plots. FLAG-mA3 indicates mice heterozygous for the KI allele. (**B**) Representative histograms showing fluorescence intensities of FLAG-tagged mA3 protein (FLAG) in each cell population of cultured spleen cells. Spleen cells from homozygous (+/+) and heterozygous (+/−) KI mice as well as those from WT B6 mice were cultured with (+) or without (−) LPS. A number of cells showing each different intensity of FLAG-specific fluorescence are shown relative to the maximum counts (% of Max) to normalize the differences in cell numbers gated into the three populations (see panel (**A**)). (**C**) Differences between genetic groups in FLAG-specific mean fluorescence intensities in each lymphoid cell subpopulation and their changes upon LPS stimulation. Mean fluorescence intensities (MFI) for intracytoplasmic FLAG staining are shown relative to the average MFI of unstimulated B6 cells for each cell population, as background staining of B6 cells differed between cell populations and was especially high for CD3^−^, CD19^−^ cells (see panel (**B**)). Relative MFI value for cells from each mouse is shown with filled symbols representing LPS stimulated and open symbols unstimulated cultures. Long and short horizontal bars overlapping with symbols indicate averages ± SEM. Group-wise differences are analyzed by one-way ANOVA with Tukey’s post-hoc tests: * *p* < 0.05; ** *p* < 0.01; *** *p* < 0.001. Experiments were performed 4 times with 2 to 5 animals for each genetic group with essentially identical results.

Our previous results indicated that mA3 mRNA is expressed rather selectively in lymphoid and hematopoietic tissues including the spleen, thymus, and bone marrow, and CD19^+^ B cells expressed higher levels of mA3 mRNA than CD3^+^ T cells in the spleen [32]. However, the possible differences in protein levels of mA3 expression and their changes upon stimulation in these lymphocyte subsets have not been examined. Thus, we next performed flow cytometric analyses of FLAG-tagged mA3 protein expression in different subpopulations of lymphoid cells using spleen cells cultured with and without LPS. After culturing in vitro for 2 days, lymphoid cells were gated, dead cells eliminated and resultant single cells were stained for CD3 and CD19 along with the anti-FLAG mAb (Figure 2A). WT B6 cells were used as negative controls for FLAG-specific staining, and the background staining levels did not increase significantly after LPS stimulation. For all three populations of CD19^+^ B, CD3^+^ T, and CD19^−^, CD3^−^ non-B, non-T cells, KI cells showed mA3 protein expression that was significantly higher in their fluorescence intensities than those of corresponding B6 cells regardless of LPS stimulation (Figure 2B,C). Importantly, expression levels of FLAG-tagged mA3 protein were significantly higher in cells homozygous for the KI allele than in heterozygous cells for all three populations. Further, in all examined populations, LPS stimulation induced significantly higher mA3 protein expression than in corresponding cells cultured without LPS. However, absolute levels of mA3 expression were the highest in CD19^+^ B cells, consistent with the previous results on mA3 mRNA expression levels [32].

### 3.3. Tissue Distribution of mA3 Protein Expression In Vivo

**Figure 3:**
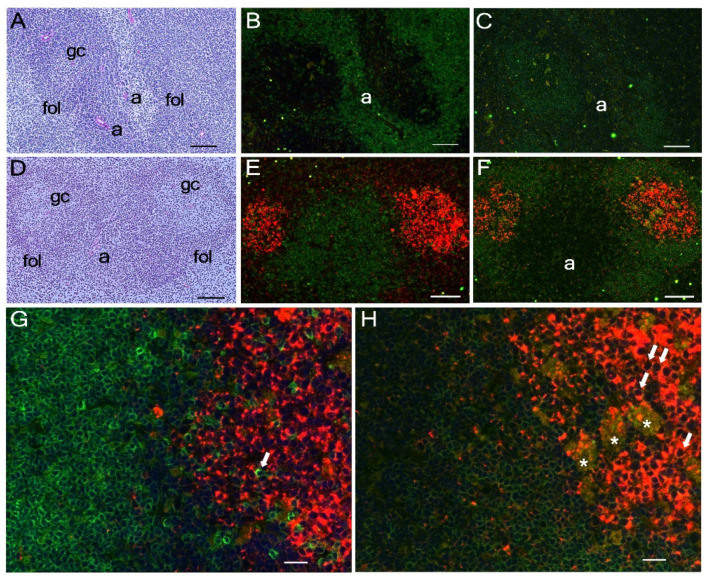
**Immunohistochemical detection of FLAG-tagged mA3 protein in the spleen.** Frozen tissue sections prepared from WT B6 (**A**–**C**) and homozygous FLAG-KI mice (**D**–**H**) were stained with hematoxylin and eosin (HE, panels (**A**,**D**)), fluorescent Ab specific for CD3 (green) and FLAG (red) (panels (**B**,**E**,**G**)) or with Ab specific for B220 (green) and FLAG (red) (panels (**C**,**F**,**H**)). Panels (**A**–**C**) and (**D**–**F**) respectively show corresponding view fields from serial sections. Bars indicate 100 μm in panels (**A**–**F**), and 20 μm in (**G**,**H**). In panels (**A**–**F**), “a” indicates a central artery, “fol” a follicle, and “gc” a germinal center within a follicle in the white pulp. Note that CD3^+^ T cells are located surrounding the central artery and B220^+^ cells are accumulated in follicles as expected. In panels (**F**,**H**) cells in GC show an orange color, while those in panels (**E**,**G**) are stained red, indicating that GC cells are positive for both B220 and FLAG. Higher magnification shows the presence of T cells within GC that are negative for FLAG (arrow in panel (**G**)), and cytoplasmic location of FLAG-tagged mA3 in GC B cells sparing nuclei (arrows in panel (**H**)). Apparent tingible body macrophages are indicated with asterisks. Immunohistochemical staining was performed a total of 7 times with a few specimens for each genetic group in each experiment with results essentially identical to those shown above.

As levels of FLAG-tagged mA3 protein expression were significantly higher in homozygotes of the KI allele than in heterozygotes, we next performed immunohistochemical analyses using tissues from KI homozygotes. No staining with the anti-FLAG Ab was observed in spleen tissues from WT B6 mice (Figure 3A–C). On the other hand, immunofluorescent staining of the spleen from homozygous KI mice showed distinctive localization of the FLAG-tagged protein in the central area of B cell follicles corresponding to germinal centers (GC), while parafollicular T-cell zones surrounding central arteries were not stained (Figure 3D–F). Higher magnifications indicated that T cells, even those in GC, were not stained with the anti-FLAG mAb (Figure 3G, arrow), while follicular B cells were co-stained for B220 and FLAG-tagged mA3 protein (see left half of Figure 3H). However, cells in GC were stained much more strongly with the anti-FLAG mAb than follicular B cells, and higher magnifications indicated that FLAG-tagged mA3 protein is located in their cytoplasm sparing nuclei (Figure 3H, arrows). It was notable that apparent tingible body macrophages contained cellular debris that was positive for B220 and FLAG-tagged mA3 protein (large greenish-yellow patches with orange speckles indicated by asterisks in Figure 3H).

**Figure 4:**
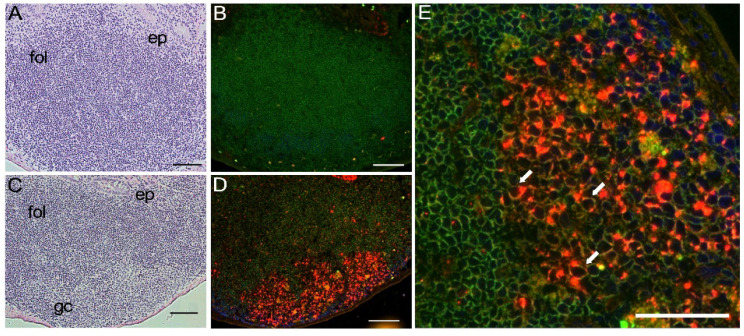
**Immunohistochemical detection of FLAG-tagged mA3 protein in Payer’s patches.** Frozen tissue sections prepared from WT B6 (**A**,**B**) and homozygous FLAG KI mice (**C**–**E**) were stained with HE (panels (**A**,**C**)) or with fluorescent Ab specific for B220 (green) and FLAG (red) (panels (**B**,**D**,**E**)). Panels (**A**,**B**) and (**C**,**D**) respectively show corresponding view fields from serial sections. Bars indicate 100 μm in panels (**A**–**D**), and 50 μm in (**E**). In panels (**A**,**C**), “ep” indicates epithelial cells, “fol” a follicle, and “gc” a germinal center within a follicle. Note that the B-cell follicle in WT B6 mouse is negative for FLAG-tagged mA3. In panels (**D**,**E**) cells in follicles and GC show an orange color, indicating that these B cells are positive for both B220 and FLAG. Higher magnification in panel (**E**) shows the cytoplasmic location of FLAG-tagged mA3 in B220^+^ large B cells sparing nuclei (arrows). Immunohistochemical staining was performed a total of 4 times with a few specimens for each genetic group in each experiment with results essentially identical to those shown above.

The above high-level expression of FLAG-tagged mA3 protein in GC prompted us to examine Peyer’s patches as these intestinal lymphoid tissues usually contain large GC, and our previous results showed a low but significant level of mA3 mRNA expression in the small intestine despite apparent lack of mA3 expression in other epithelial tissues [32]. Immunohistochemical analyses of Peyer’s patches revealed very high levels of FLAG-tagged mA3 protein expression in GC of homozygous KI mice, while no significant staining with the anti-FLAG mAb was observed in control B6 tissues (Figure 4). Again, co-staining for B220 and FLAG-tagged mA3 protein was observed in large B220^+^ B cells, but nuclei were not stained (Figure 4E, arrows).

### 3.4. Flow Cytometric Analyses of B-Cell Subpopulations for Their mA3 Protein Expression before and after In Vivo Immunization with Sheep Red Blood Cells

**Figure 5:**
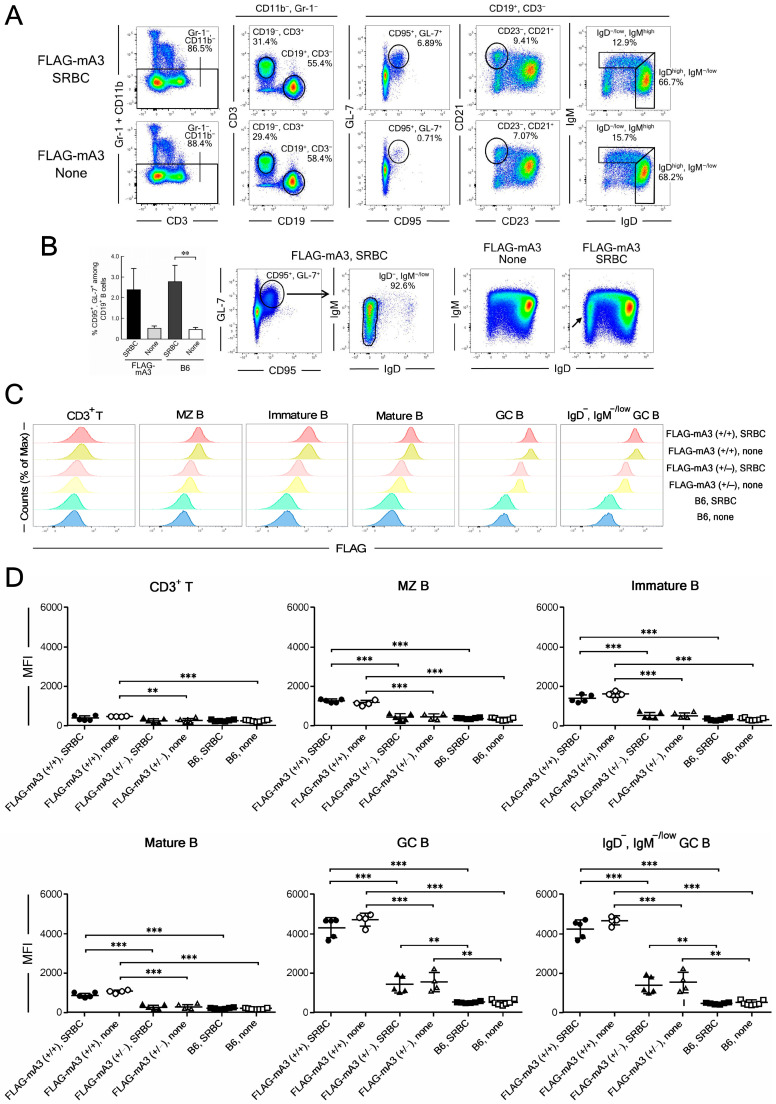
**Flow cytometric analyses of intracytoplasmic mA3 expression in different subpopulations of spleen cells before and after immunization with SRBC in vivo.** (**A**) Representative gating strategies were shown for cells from heterozygous FLAG-KI animals before (none) and 10 days after SRBC injection. Mononuclear cells were gated from freshly prepared spleen cells by using dot plots showing FSC-A and SSC-A and dead cells eliminated by staining with FVS780 similarly to those shown in Figure 2, and doublets were gated out based on comparisons of signal width and height parameters of FSC and SSC according to the standard procedure (BD Biosciences). To analyze lymphoid cells alone, myeloid cells were eliminated by staining with anti-Gr-1 and anti-CD11b Ab, and CD19^+^ B and CD3^+^ T cells were gated from the above lymphoid cells by using dot plots showing fluorescence intensities of CD3 and CD19. CD19^+^ B cells were further analyzed for their expression of CD95 (Fas), GL-7, CD21, CD23, surface (s) IgM, and sIgD. To make the gate settings more accurate, 100,000 events were used from the obtained 1,000,000 to generate down-sampled dot plots shown in panel (**A**). (**B**) Changes in numbers and phenotypes of CD95^+^, GL-7^+^ GC cells. Percentages of CD95^+^, GL-7^+^ GC cells among CD19^+^ B cells were determined as shown in panel (**A**) and averages were calculated for heterozygous FLAG KI and WT B6 groups both before (none) and after immunization with SRBC. Bars in the leftmost chart indicate mean + SEM. **, *p* = 0.0092 by two-tailed Student’s *t*-test. Dot plots in the middle two charts of the panel (**B**) show representative patterns of sIgM and sIgD expression on CD95^+^, GL-7^+^ GC B cells. Most of these CD95^+^, GL-7^+^ GC B cells were sIgD^−^ and showed low to negative sIgM expression. These sIgD^−^ GC B cells were gated as shown in the central chart and analyzed for their expression of cytoplasmic FLAG-tagged mA3 as presented in panels (**C**,**D**) Comparison of sIgM/sIgD dot plots between unimmunized (none) and SRBC-immunized animals as shown in the right side of the panel (**B**) for CD19^+^ B cells revealed obvious increase in sIgD^−^, sIgM^−/low^ cell population after immunization (arrow). Unlike those in panel (**A**), dot plots in panel (**B**) are generated with the entire 1,000,000 events for each animal. (**C**) Representative histograms showing fluorescence intensities of FLAG-tagged mA3 protein (FLAG) in each subpopulation of B cells. Spleen cells from homozygous (+/+) and heterozygous (+/−) KI mice as well as those from WT B6 mice were prepared before (none) and 10 days after SRBC injection. The number of cells exhibiting each different intensity of FLAG-specific fluorescence is shown relative to the maximum counts (% of Max) to normalize the differences in cell numbers gated into the T and B cell subpopulations (see panel (**A**)). (**D**) Differences between genetic groups in FLAG-specific mean fluorescence intensities (MFI) in each lymphoid cell subpopulation and their changes upon SRBC injection. Absolute MFI value for cells from each mouse is shown with filled symbols representing SRBC stimulated and open symbols unimmunized (none) mice. Long and short horizontal bars overlapping with symbols indicate averages ± SEM. Group-wise differences are analyzed by one-way ANOVA with Tukey’s post-hoc tests: ** *p* < 0.01; *** *p* < 0.001. Experiments were performed 3 times with 4 to 7 animals for each genetic group with essentially identical results.

To examine in more detail the presumed differences in mA3 protein expression in separate subpopulations of B cells, we next performed flow cytometric analyses using spleen cells. Mice were injected with viable SRBC which is known to result in increased numbers and sizes of GC in the spleen without using any adjuvant [38]. Spleen cells were prepared from SRBC-injected and non-injected animals, myeloid cells eliminated from live single cells by staining with anti-CD11b and anti-Gr-1 Ab, and resultant lymphoid cells were first separated into CD3^+^ T and CD19^+^ B cell populations (Figure 5A). CD19^+^ B cells were further separated into CD95^+^, GL-7^+^ GC B cells, and these GC B cells were examined for their expression of cell-surface IgD and IgM (Figure 5B). As expected, percentages of CD95^+^, GL-7^+^ GC B cells increased after SRBC injection (Figure 5B). The vast majority of these CD95^+^, GL-7^+^ cells were IgD-negative and negative to low in their expression of surface IgM, consistent with previous reports [39,40]. As the spleen is the site of B-cell maturation and separate stages of maturing B cells may be differentially affected by retrovirus infection [32], we also separated CD19^+^ B cells into IgM^high^, IgD^−/low^ immature B cells, IgD^high^ mature B cells and CD23^−^, CD21^+^ marginal zone (MZ) B cells (Figure 5A), and examined their expression of FLAG-tagged mA3 protein. In consistency with the cell surface phenotypes of GC B cells, IgD^−^, IgM^−/low^ population was increased upon SRBC injection (Figure 5B, IgM/IgD plots).

Although cultured T cells expressed low levels of mA3 that was increased after LPS stimulation (Figure 2C), freshly prepared spleen T cells showed merely background levels of FLAG-tagged mA3 protein expression that reached significantly higher than those in control B6 cells only in non-stimulated homozygotes for the KI allele, but the difference did not reach statistical significance after SRBC stimulation (Figure 5C,D). Thus, physiological levels of mA3 protein expression in spleen T cells were very low, consistent with the results of immunohistochemical analyses (Figure 3G). On the other hand, B cells of all examined differentiation and activation stages in homozygous KI mice showed mA3 expression levels that were significantly higher than those in heterozygous and WT animals (Figure 5D). This is consistent with the costaining of B220 and FLAG signals in follicular B cells in homozygous KI mice revealed by immunohistochemical approaches (Figure 3 and Figure 4). Importantly, CD95^+^, GL-7^+^ GC B cells in homozygous as well as heterozygous KI mice showed high levels of FLAG-tagged mA3 protein expression that were significantly more intense than background levels observed in control WT B6 cells. The same levels of FLAG-tagged mA3 protein expression were observed when GC B cells were further selected for their lack of cell-surface IgD expression (Figure 5C,D). FLAG-tagged mA3 protein expression at significantly higher levels than those in WT B6 mice was observed only for GC B cells but not for other B-cell subpopulations in heterozygous mice indicating, along with absolutely high fluorescence intensities in GC B cells, that GC B cells express distinctively high levels of mA3 protein among different B-cell differentiation stages. Further, no significant differences in FLAG-tagged mA3 protein expression levels were observed between SRBC-stimulated and non-stimulated animals of the same genotype despite increased numbers of GC B cells upon SRBC injection. Thus, it can be concluded that the above distinctive high expression of mA3 protein depends on the differentiation stage of B cells, and the antigenic stimulation increases the number of mA3^high^ GC B cells without affecting levels of mA3 protein expression in each cell.

## 4. Discussion

While the human *APOBEC3* gene locus contains 7 paralogues, mice possess a single *Apobec3* gene on chromosome 15, of which several different alleles associated with spontaneous resistance or susceptibility to mouse retroviral infections are described [3,8,9,11]. These mA3 alleles are codominantly expressed in heterozygous individuals [9,11], resulting in the presence of two transcripts with distinct translational efficiencies [13]. Thus, it is reasonable that FLAG-tagged mA3 protein was detected in significantly higher levels in homozygous than in heterozygous KI mice in our experiments.

Our initial examination using cultured spleen cells showed high levels of mA3 protein expression in B cells that increased significantly after LPS stimulation, while mA3 protein expression in T cells was low. These results were consistent with previous findings that B cells express higher levels of mA3 mRNA than T cells [32]. Interestingly, CD19^−^, CD3^−^ cells expressed intermediate levels of mA3 protein that were significantly increased after LPS stimulation (Figure 2C). It has been shown that mouse monocyte-derived dendritic cells express high levels of mA3 after LPS stimulation [36,37] and thus mA3-expressing non-B, non-T cells we have observed may include monocytes or dendritic cell precursors. In addition, human NK cells are known to express APOBEC3 messages at higher levels than T- and B-lymphocytes or myeloid cells [41]. It has not been examined if mouse NK cells express mA3; however, as NK cells are known to express TLR4 [42], they can respond to LPS stimulation and increase mA3 protein expression. Further studies are required to identify the nature of CD19^−^, CD3^−^ cells that express mA3 protein and the possible physiological roles of mA3 in these cells.

Previous analyses using human tissues have indicated that APOBEC3 proteins are detectable in the GC of the adenoid and palatine tonsils [20]. However, cell populations that express APOBEC3 proteins in human GC were not identified in detail. Our FLAG-KI mice not only made it possible to demonstrate the localization of FLAG-tagged mA3 protein in GC but also to identify CD95^+^, GL-7^+^ GC B cells as well as their IgD^−^ subpopulation as the B-cell differentiation stage at which distinctively high levels of mA3 protein is expressed. Physiological roles of mA3 protein in GC B cells are currently unknown; however, B cells have long been implicated as a reservoir of persistent Friend retrovirus infection [43] and recent analyses utilizing a newly developed fluorescent virus revealed that follicular B cells are initially infected with FV in the acute phase and the infection persisted in IgM^−^, IgD^−^ class-switched B cells in late phase [44]. It is conceivable that infected precursor cells go through proliferative burst in GC, resulting in apparent persistence of provirus-possessing cells among GC B cells and their long-living progenies, rather than GC B cells being newly infected in the chronic phase. Thus, distinctive high expression of mA3 protein in GC B cells including IgD^−^, IgM^−/low^ population can be pertinent to the known physiological function of mA3 in restricting FV infection in vivo [9]. 

It is of note that the above genetic polymorphisms at the mouse *Apobec3* locus have been associated with the rate of production of virus-neutralizing Ab upon FV infection [8,9]. Although it has been shown that AID-mediated somatic hypermutation is not required for the generation of FV-neutralizing Ab and that non-mutated IgM Ab can still neutralize FV infectivity [45], mA3 has been implicated in the induction of higher levels of somatic hypermutation in the immunoglobulin gene variable regions associated with stronger binding of resultant Ab to native viral proteins [46]. To induce somatic hypermutation, mA3, if involved, must bind genomic DNA, and AID has been shown to colocalize with chromatin structures in dividing cells [47]. On the other hand, despite active nuclear importation and steady-state localization in cellular nuclei, human A3B does not colocalize with chromosomes during mitosis, and enforced A3B expression cannot substitute for AID in immunoglobulin gene diversification by class switch recombination [48]. It has also been shown that human A3A, A3C, and A3H are excluded from condensed chromatins despite their cell-wide distribution during telophase, and A3B, A3D, A3F, and A3G are excluded from chromatin structures throughout mitoses [47]. Thus, the apparent lack in nuclear localization of FLAG-tagged mA3 protein in GC B cells despite its high expression levels might indicate that similarly to human A3B, mA3 is unlikely to substitute for AID in the induction of immunoglobulin gene diversification. To examine more precisely the possibility that mA3 protein might be transiently attached to genomic DNA in dividing GC B cells, subcellular localization analyses with higher resolution are required. 

While human splenic MZ B cells were negative for AID expression [49], our data have indicated that mA3 protein is expressed in splenic MZ B cells as well as maturing B-cell precursors at low but significant levels. Presumable species-specific differences aside, a more confined distribution of AID compared with that of APOBEC3 proteins is reasonable when considering the possible deleterious effect on genomic DNA of nuclear localizing AID [14,15,16]. 

Although antigenic stimulation with SRBC in vivo did not change the mA3 protein expression levels in each B cell despite an obvious increase in GC cell numbers, in vitro stimulation of spleen cells with LPS resulted in significantly increased expression of mA3 protein in B cells. These differences may reflect distinct effects of separate signaling pathways, namely those mediated through T-B cell interactions and TLR-4-mediated, in regulating the *Apobec3* gene expression, which has been reported for IFN-α and LPS [37]. Thus, our KI model would be useful in investigating specific inflammatory and immune stimulations as well as those associated with carcinogenesis that change APOBEC3 protein expression levels and subcellular localization in hematopoietic, epithelial, and other cell types. 

## 5. Conclusions

FLAG KI animals revealed distinctive high expression levels of mA3 protein in the cytoplasm of GC B cells. The high expression level is specific for this differentiation stage of B cells, and antigenic stimulation does not affect mA3 protein levels in each cell despite the increased number of GC cells. As B cells are known initial targets of Friend retrovirus infection, these findings are pertinent to the physiological function of mA3 as a retrovirus-restricting factor.

## Supplementary Materials

The following are available online at https://www.mdpi.com/article/10.3390/v14040832/s1. Figure S1: Genomic sequence around exon 1 of the Apobec3 gene. Figure S2: Photographs of the entire agarose gel used to generate Figure 1, panel B. Figure S3: Photographs of the entire membrane used to generate Figure 1, panel C. Figure S4: A photograph of the entire membrane used to generate Figure 1, panel D. Figure S5: Sequence analysis of the entire coding region of *Apobec3* cDNA cloned from E1 spleen cells.
