# Supplementary material for: Distinctive High Expression of Antiretroviral APOBEC3 Protein in Mouse Germinal Center B Cells
Source: Viruses. 2022 Apr 17;14(4):832. doi: 10.3390/v14040832 (PMC9029289; doi:10.3390/v14040832)
Supplement: Supplementary file 1 [file viruses-14-00832-s001.zip › Figure S5.pdf]

```
                           FLAG
E1_FLAG-mA3   ATGGACTACAAAGACGATGACGACAAGGGACCATTCTGTCTGGGATGCAGCCATCGCAAA
isoform_2     ------------------------ATGGGACCATTCTGTCTGGGATGCAGCCATCGCAAA
isoform_1     ------------------------ATGGGACCATTCTGTCTGGGATGCAGCCATCGCAAA
                                      *:**********************************

E1_FLAG-mA3   TGCTATTCACCGATCAGAAACCTGATATCTCAAGAAACATTCAAGTTCCACTTTAAGAAC
isoform_2     TGCTATTCACCGATCAGAAACCTGATATCTCAAGAAACATTCAAGTTCCACTTTAAGAAC
isoform_1     TGCTATTCACCGATCAGAAACCTGATATCTCAAGAAACATTCAAGTTCCACTTTAAGAAC
              ************************************************************

E1_FLAG-mA3   CTAGGCTATGCCAAAGGCCGGAAAGATACCTTCTTGTGCTATGAAGTGACTAGAAAGGAC
isoform_2     CTAGGCTATGCCAAAGGCCGGAAAGATACCTTCTTGTGCTATGAAGTGACTAGAAAGGAC
isoform_1     CTAGGCTATGCCAAAGGCCGGAAAGATACCTTCTTGTGCTATGAAGTGACTAGAAAGGAC
              ************************************************************

E1_FLAG-mA3   TGCGATTCACCCGTCTCCCTTCACCATGGGGTCTTTAAGAACAAGGACAACATCCACGCT
isoform_2     TGCGATTCACCCGTCTCCCTTCACCATGGGGTCTTTAAGAACAAGGACAACATCCACGCT
isoform_1     TGCGATTCACCCGTCTCCCTTCACCATGGGGTCTTTAAGAACAAGGACAACATCCACGCT
              ************************************************************

E1_FLAG-mA3   GAAATCTGCTTTTTATACTGGTTCCATGACAAAGTACTGAAAGTGCTGTCTCCGAGAGAA
isoform_2     GAAATCTGCTTTTTATACTGGTTCCATGACAAAGTACTGAAAGTGCTGTCTCCGAGAGAA
isoform_1     GAAATCTGCTTTTTATACTGGTTCCATGACAAAGTACTGAAAGTGCTGTCTCCGAGAGAA
              ************************************************************

E1_FLAG-mA3   GAGTTCAAGATCACCTGGTATATGTCCTGGAGCCCCTGTTTCGAATGTGCAGAGCAGATA
isoform_2     GAGTTCAAGATCACCTGGTATATGTCCTGGAGCCCCTGTTTCGAATGTGCAGAGCAGATA
isoform_1     GAGTTCAAGATCACCTGGTATATGTCCTGGAGCCCCTGTTTCGAATGTGCAGAGCAGATA
              ************************************************************

E1_FLAG-mA3   GTAAGGTTCCTGGCTACACACCACAACCTGAGCCTGGACATCTTCAGCTCCCGCCTCTAC
isoform_2     GTAAGGTTCCTGGCTACACACCACAACCTGAGCCTGGACATCTTCAGCTCCCGCCTCTAC
isoform_1     GTAAGGTTCCTGGCTACACACCACAACCTGAGCCTGGACATCTTCAGCTCCCGCCTCTAC
              ************************************************************

E1_FLAG-mA3   AACGTACAGGACCCAGAAACCCAGCAGAATCTTTGCAGGCTGGTTCAGGAAGGAGCCCAG
isoform_2     AACGTACAGGACCCAGAAACCCAGCAGAATCTTTGCAGGCTGGTTCAGGAAGGAGCCCAG
isoform_1     AACGTACAGGACCCAGAAACCCAGCAGAATCTTTGCAGGCTGGTTCAGGAAGGAGCCCAG
              ************************************************************
```

```
E1_FLAG-mA3   GTGGCTGCCATGGACCTATACGAATTTAAAAAGTGTTGGAAGAAGTTTGTGGACAATGGT
isoform_2     GTGGCTGCCATGGACCTATACGAATTTAAAAAGTGTTGGAAGAAGTTTGTGGACAATGGT
isoform_1     GTGGCTGCCATGGACCTATACGAATTTAAAAAGTGTTGGAAGAAGTTTGTGGACAATGGT
              ************************************************************

E1_FLAG-mA3   GGCAGGCGATTCAGGCCTTGGAAAAGACTGCTTACAAATTTTAGATACCAGGATTCTAAG
isoform_2     GGCAGGCGATTCAGGCCTTGGAAAAGACTGCTTACAAATTTTAGATACCAGGATTCTAAG
isoform_1     GGCAGGCGATTCAGGCCTTGGAAAAGACTGCTTACAAATTTTAGATACCAGGATTCTAAG
              ************************************************************

E1_FLAG-mA3   CTTCAGGAGATTCTGAG-------------------------------------------
isoform_2     CTTCAGGAGATTCTGAG-------------------------------------------
isoform_1     CTTCAGGAGATTCTGAGACCTTGCTACATCTCGGTCCCTTCCAGCTCTTCATCCACTCTG
              *****************

E1_FLAG-mA3   --------------------------------------------------------GCGA
isoform_2     --------------------------------------------------------GCGA
isoform_1     TCAAATATCTGTCTAACAAAAGGTCTCCCAGAGACGAGGTTCTGGGTGGAGGGCAGGCGA
                                                                      ****

E1_FLAG-mA3   ATGGACCCGCTAAGTGAAGAGGAATTTTACTCGCAGTTTTACAACCAACGAGTCAAGCAT
isoform_2     ATGGACCCGCTAAGTGAAGAGGAATTTTACTCGCAGTTTTACAACCAACGAGTCAAGCAT
isoform_1     ATGGACCCGCTAAGTGAAGAGGAATTTTACTCGCAGTTTTACAACCAACGAGTCAAGCAT
              ************************************************************

E1_FLAG-mA3   CTCTGCTACTACCACCGCATGAAGCCCTATCTATGCTACCAGCTGGAGCAGTTCAATGGC
isoform_2     CTCTGCTACTACCACCGCATGAAGCCCTATCTATGCTACCAGCTGGAGCAGTTCAATGGC
isoform_1     CTCTGCTACTACCACCGCATGAAGCCCTATCTATGCTACCAGCTGGAGCAGTTCAATGGC
              ************************************************************

E1_FLAG-mA3   CAAGCGCCACTCAAAGGCTGCCTGCTAAGCGAGAAAGGCAAACAGCATGCAGAAATCCTC
isoform_2     CAAGCGCCACTCAAAGGCTGCCTGCTAAGCGAGAAAGGCAAACAGCATGCAGAAATCCTC
isoform_1     CAAGCGCCACTCAAAGGCTGCCTGCTAAGCGAGAAAGGCAAACAGCATGCAGAAATCCTC
              ************************************************************

E1_FLAG-mA3   TTCCTTGATAAGATTCGGTCCATGGAGCTGAGCCAAGTGACAATCACCTGCTACCTCACC
isoform_2     TTCCTTGATAAGATTCGGTCCATGGAGCTGAGCCAAGTGACAATCACCTGCTACCTCACC
isoform_1     TTCCTTGATAAGATTCGGTCCATGGAGCTGAGCCAAGTGACAATCACCTGCTACCTCACC
              ************************************************************
```

```
E1_FLAG-mA3   TGGAGCCCCTGCCCAAACTGTGCCTGGCAACTGGCGGCATTCAAAAGGGATCGTCCAGAT
isoform_2     TGGAGCCCCTGCCCAAACTGTGCCTGGCAACTGGCGGCATTCAAAAGGGATCGTCCAGAT
isoform_1     TGGAGCCCCTGCCCAAACTGTGCCTGGCAACTGGCGGCATTCAAAAGGGATCGTCCAGAT
              ************************************************************

E1_FLAG-mA3   CTAATTCTGCATATCTACACCTCCCGCCTGTATTTCCACTGGAAGAGGCCCTTCCAGAAG
isoform_2     CTAATTCTGCATATCTACACCTCCCGCCTGTATTTCCACTGGAAGAGGCCCTTCCAGAAG
isoform_1     CTAATTCTGCATATCTACACCTCCCGCCTGTATTTCCACTGGAAGAGGCCCTTCCAGAAG
              ************************************************************

E1_FLAG-mA3   GGGCTGTGTTCTCTGTGGCAATCAGGGATCCTGGTGGACGTCATGGACCTCCCACAGTTT
isoform_2     GGGCTGTGTTCTCTGTGGCAATCAGGGATCCTGGTGGACGTCATGGACCTCCCACAGTTT
isoform_1     GGGCTGTGTTCTCTGTGGCAATCAGGGATCCTGGTGGACGTCATGGACCTCCCACAGTTT
              ************************************************************

E1_FLAG-mA3   ACTGACTGCTGGACAAACTTTGTGAACCCGAAAAGGCCGTTTTGGCCATGGAAAGGATTG
isoform_2     ACTGACTGCTGGACAAACTTTGTGAACCCGAAAAGGCCGTTTTGGCCATGGAAAGGATTG
isoform_1     ACTGACTGCTGGACAAACTTTGTGAACCCGAAAAGGCCGTTTTGGCCATGGAAAGGATTG
              ************************************************************

E1_FLAG-mA3   GAGATAATCAGCAGGCGCACACAAAGGCGGCTCCGCAGGATCAAGGAGTCCTGGGGTCTG
isoform_2     GAGATAATCAGCAGGCGCACACAAAGGCGGCTCCGCAGGATCAAGGAGTCCTGGGGTCTG
isoform_1     GAGATAATCAGCAGGCGCACACAAAGGCGGCTCCGCAGGATCAAGGAGTCCTGGGGTCTG
              ************************************************************

E1_FLAG-mA3   CAAGATTTGGTGAATGACTTTGGAAACCTACAGCTTGGACCCCCGATGTCTTGA
isoform_2     CAAGATTTGGTGAATGACTTTGGAAACCTACAGCTTGGACCCCCGATGTCTTGA
isoform_1     CAAGATTTGGTGAATGACTTTGGAAACCTACAGCTTGGACCCCCGATGTCTTGA
              ******************************************************
```

**Figure S5. Sequence analysis of the entire coding region of *Apobec3* cDNA cloned from E1 spleen cells.** Spleen cell mRNA was prepared from E1 mouse, reverse transcribed as described for Figure S2, and the *Apobec3* cDNA was amplified by using primers specific for 5' and 3' UTRs of the FLAG-tagged mA3 allele (5'-CAGAAAATGCAACCCCAGCGC-3' and 5'-CATGCACAACTTAATCTTGTCTTTC-3', respectively) and Pfu Turbo DNA Polymerase (Agilent Technologies, Santa Clara, California, U.S.A.). The amplified fragment was cloned into pCR-Blunt II-TOPO plasmid using the Zero Blunt TOPO PCR Cloning Kit (Invitorogen, Thermo Fisher Scientific) and sequenced as described [9]. The resultant DNA sequence was aligned with the consensus isoform 1 (NCBI Reference Sequence: NM_001160415.1) and isoform 2 (NCBI Reference Sequence: NM_030255.3) sequences using the Clustal W (https://www.genome.jp/tools-bin/clustalw).
