# Supplementary material for: Distinctive High Expression of Antiretroviral APOBEC3 Protein in Mouse Germinal Center B Cells
Source: Viruses. 2022 Apr 17;14(4):832. doi: 10.3390/v14040832 (PMC9029289; doi:10.3390/v14040832)
Supplement: Supplementary file 1 [file viruses-14-00832-s001.zip › Figure S1.pdf]

Acctgctgctggctgattcggagccatcaatgatccatttttgtgctccccgttgatctcagtctagctccacacctggggcagtt
gggaaattagaggtgaagggtgccaaaatccaagccacaattagaaatgcgaagccaggctgtcccctcccctcctaagccg
aagggagaaaatgtaagggtccccactccttaacacctacagagtcaaacccttggttcaactggcagctttggctgcaagct
cggcacaggatggtctaggcaggttggatggagacgcctggggccacccttggggcgtctgtcctaccctcaccgcggtcggc
atcagcttctctctaccatcagttagtttccctttctctttgcgatccccgcgggccgctgtacagaggagcaggtctctgccaag
ccaagggacaaagaggtgggcaggagccttctgggcttgatcgcctagagccctcccattccgggagctgatgcaggaag*A*
AGGTGGGGCCTGCATTCACTTGGCCCGGGAGGTCAGTTTCACTTCTGGGGTCTT
*CCATAGCCTGCTCACAGAAAATGCAACCCCAGCGCCTGGGGCCCAGAGCTGGG***AT**
GGACTACAAAGACGATGACGACAAG**GGACCATTCTGTCTGGGATGCAGCCATCGC**
AAATGCTATTCACCGATCAGgtaccgctggccccgctcctccaaaattttcactgcagtctcttcctggtggccccc
attttgctgtataccgagcctctcctctaggtccccagctcccgattcctctctcacattctccaggatactccgtagctcccactcc
cctcgagggtccttgttctgagccgcttcggttaccttatagctgcttctaaactggccaccctgtacccctctccacgtcacccat
atttcttcctgttgagacttctcagtctggggttactcgcctttgggtcttcatgctgatccagggctaggctggtctgggcttcaa
attttaaagagaaatccatggaagcagactttggacgaccatgtatggagaactttcgatagatgtcctagaatatccgtgct
gttttaacccaacctcaggggatactgggagaagaggggaccactctctaaggcaatgtgaaccacctttccagaagtagcc
actctgagctgggaaa

Figure S1. Genomic sequence around exon 1 of the *Apobec3* gene. Exon 1 including the UTR (italicized) and the wild-type CDS (bold) is highlighted with yellow background color, inserted FLAG sequence is shown in red, the CRISPR target sequences are boxed, and ssODN sequence underlined.
