# Supplementary material for: Distinctive High Expression of Antiretroviral APOBEC3 Protein in Mouse Germinal Center B Cells
Source: Viruses. 2022 Apr 17;14(4):832. doi: 10.3390/v14040832 (PMC9029289; doi:10.3390/v14040832)
Supplement: Supplementary file 1 [file viruses-14-00832-s001.zip › Figure S2.pdf]

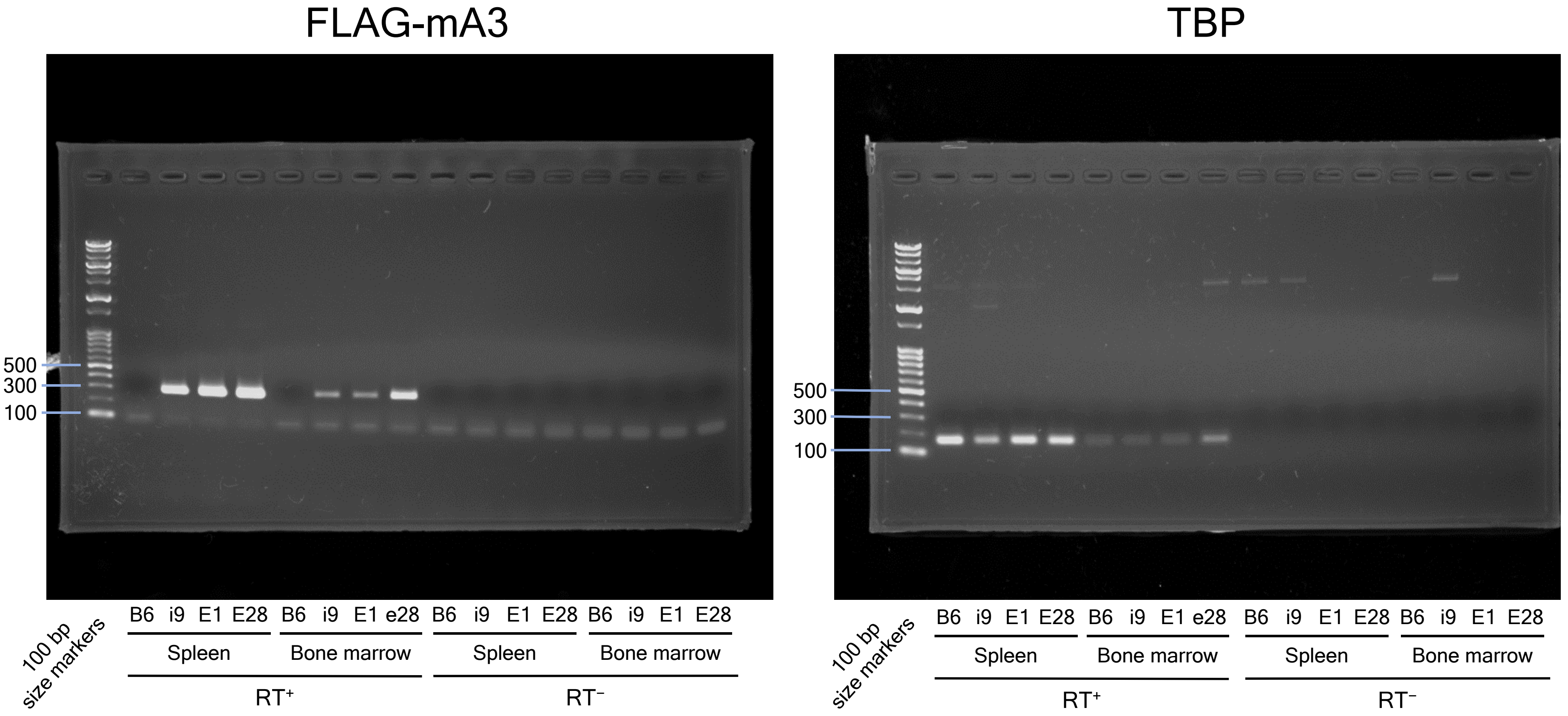

Figure S2. Photographs of the entire agarose gel used to generate Figure 1, panel B. RNA was extracted from spleen and bone marrow cells prepared from indicated lines of mice as described in the Materials and Methods section of the text and reverse transcribed with SuperScript III First-Strand Synthesis System for RT-PCR (Invitrogen, Thermo Fisher Scientific). RNA samples prior to (RT^{-}) and after (RT^{+}) reverse transcription were amplified by using Taq DNA polymerase with each of the primer sets specific for FLAG-tagged mA3 (FLAG-mA3) or TATA-binding protein (TBP). Agarose gel electrophoresis and detection of amplified fragments were done as described previously [9, 13].
