# Supplementary material for: Distinctive High Expression of Antiretroviral APOBEC3 Protein in Mouse Germinal Center B Cells
Source: Viruses. 2022 Apr 17;14(4):832. doi: 10.3390/v14040832 (PMC9029289; doi:10.3390/v14040832)
Supplement: Supplementary file 1 [file viruses-14-00832-s001.zip › Figure S3.pdf]

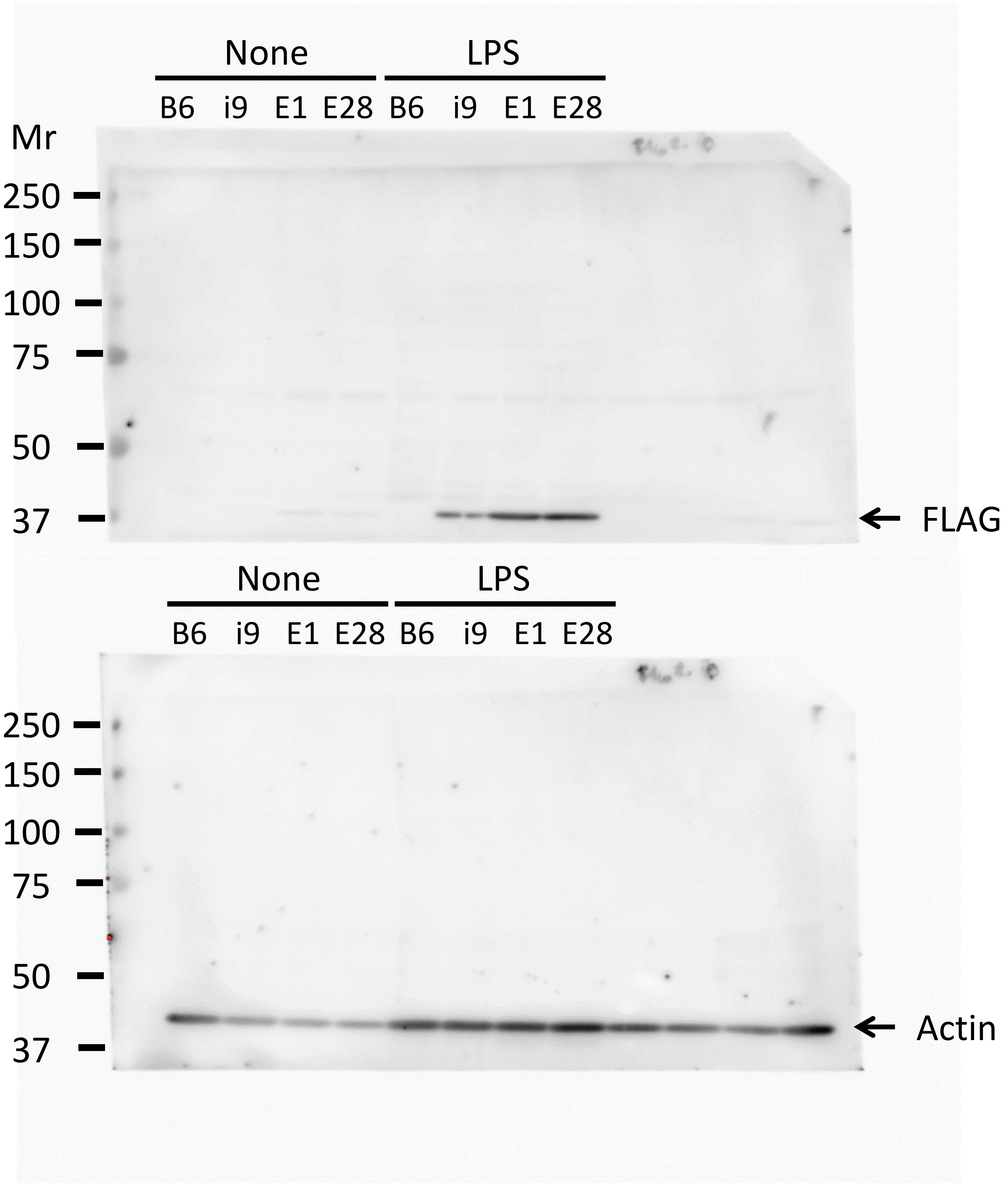

Figure S3. Photographs of the entire membrane used to generate Figure 1, panel C. Spleen cells from indicated lines of mice were cultured for two days by adding PBS alone (none) or PBS containing LPS, and their proteins were dissolved in the lysis buffer and diluted with SDS-PAGE sample buffer. After electrophoresis, blotted proteins were probed with the anti-FLAG or anti-actin Ab. Right 4 lanes are irrelevant to the present study. Mr, relative molecular mass (kDa).
