# Supplementary material for: Distinctive High Expression of Antiretroviral APOBEC3 Protein in Mouse Germinal Center B Cells
Source: Viruses. 2022 Apr 17;14(4):832. doi: 10.3390/v14040832 (PMC9029289; doi:10.3390/v14040832)
Supplement: Supplementary file 1 [file viruses-14-00832-s001.zip › Figure S4.pdf]

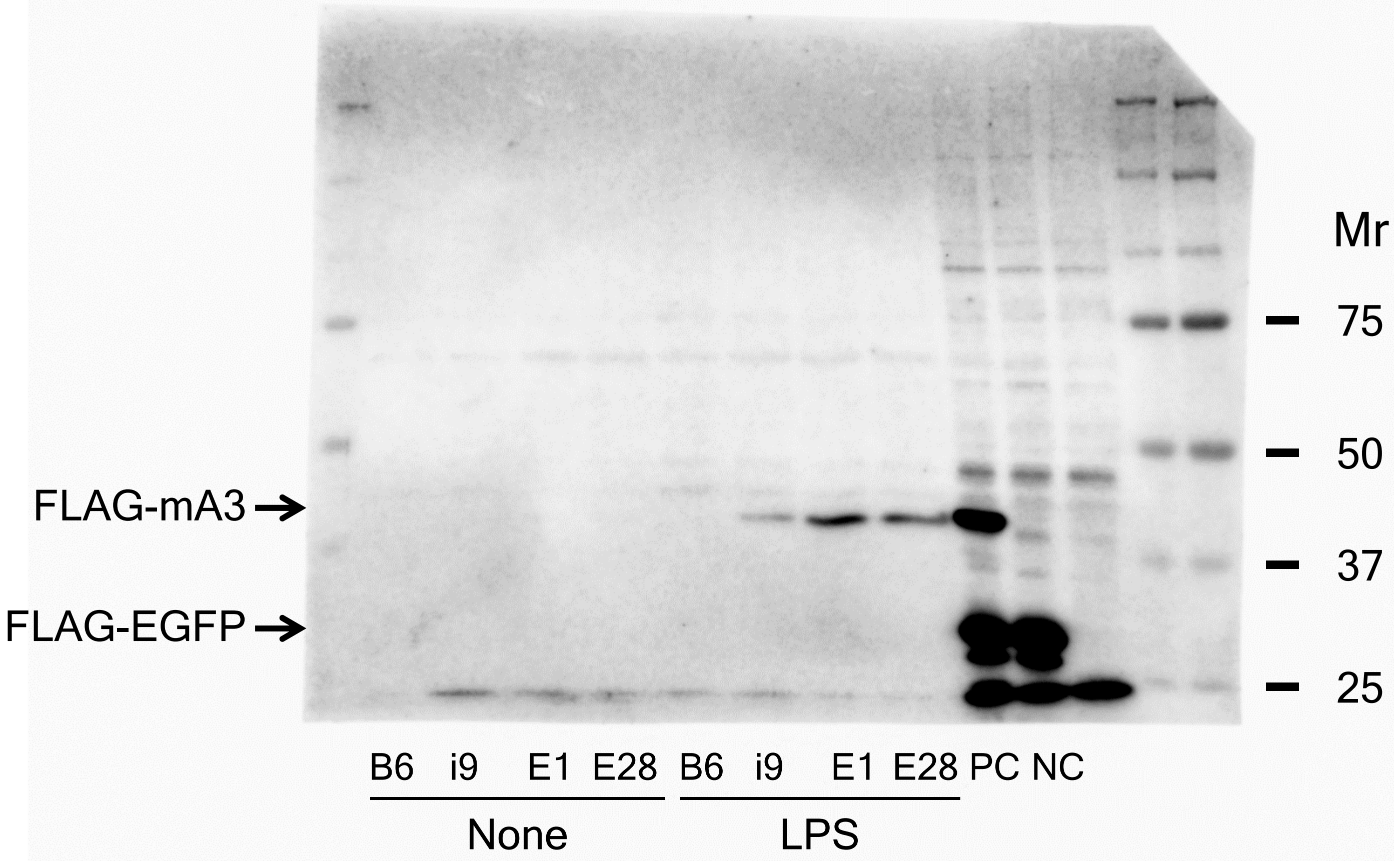


**Figure S4. A photograph of the entire membrane used to generate Figure 1, panel D.** Spleen cells from indicated lines of mice were cultured for two days by adding PBS alone (none) or PBS containing LPS, and their proteins were dissolved in the lysis buffer and diluted with SDS-PAGE sample buffer. After electrophoresis, blotted proteins were probed with the anti-FLAG Ab. As positive and negative controls, 293T cells transfected either with the pFLAG-CMV-2 plasmid expressing the full-length mA3 (isoform 2) cDNA (PC) or those transfected with the empty pFLAG-CMV-2 (NC) [26] were included. pFLAG-CMV-2 expressing EGFP was used to normalize transfection efficiencies, and thus this protein is detected from both PC and NC cells. Mr, relative molecular mass (kDa).
